# A survey of five *Pireneitega* species (Agelenidae, Coelotinae) from China

**DOI:** 10.3897/zookeys.663.11356

**Published:** 2017-03-27

**Authors:** Xiaoqing Zhang, Zhe Zhao, Guo Zheng, Shuqiang Li

**Affiliations:** 1 College of Life Science, Shenyang Normal University, Shenyang, Liaoning 110034, China; 2 Southeast Asia Biodiversity Research Institute, Chinese Academy of Sciences, Yezin, Nay Pyi Taw 05282, Myanmar; 3 Institute of Zoology, Chinese Academy of Sciences, Beijing 100101, China; 4 University of Chinese Academy of Sciences, Beijing 100049, China

**Keywords:** Taxonomy, description, diagnosis, East Asia, *Paracoelotes*

## Abstract

Five species of *Pireneitega* spiders from China are surveyed, of which three are new to science: *P. huashanensis* Zhao & Li, **sp. n.** (♂♀), *P. lushuiensis* Zhao & Li, **sp. n.** (♂♀), *P. xiyankouensis* Zhao & Li, **sp. n.** (♂♀). Two known species are redescribed: *P. liansui* (Bao & Yin, 2004) and *P. triglochinata* (Zhu & Wang, 1991). The males of *P. liansui* and *P. triglochinata* (Zhu & Wang, 1991) are described for the first time. DNA barcodes for five species are documented for future use and as proof of molecular differences between species.

## Introduction

Coelotinae is the largest subfamily of Agelenidae, with 25 genera and 676 valid species distributed in the Holarctic and southeast Asia (World Spider Catalog 2017). The genus *Pireneitega* Kishida, 1955 is one of the most species-rich genera of the subfamily. Thirty-five valid *Pireneitega* species are distributed from Europe to East Asia (Zhang and Marusik 2016), and 20 were known from China before the current study (Li and Lin 2016; Zhang et al. 2016).

During the study of *Pireneitega* spiders from China, five interesting species were found. The goal of this paper is to provide descriptions of three new species and redescriptions of two poorly known species.

## Material and methods

Specimens were examined with a Leica M205C stereomicroscope. Images were captured with an Olympus C7070 wide zoom digital camera (7.1 megapixels) mounted on an Olympus SZX12 dissecting microscope. Epigynes and male palps were examined after dissection from the spiders’ bodies. The epigyne was cleared by boiling it in 10% KOH solution before taking photos of the vulva. All measurements were obtained using a Leica M205C stereomicroscope and are given in millimeters. Leg measurements are given as: Total length (femur, patella + tibia, metatarsus, tarsus). Only structures (palp and legs) of the left side of the body are described and measured.

Terminology used for copulatory organ characters in the text and figure legends follows Wang (2002) with some modifications. Abbreviations used in the text and figure legends are: A = epigynal atrium; ALE = anterior lateral eye; AME = anterior median eye; AME-ALE = distance between AME and ALE; AME-AME = distance between AME and AME; ALE-PLE = distance between ALE and PLE; CD = copulatory ducts; CF = cymbial furrow; CO = conductor; d = dorsal; E = embolus; EB = embolic base; ET = epigynal teeth; FD = fertilization ducts; Fe = femur; H = epigynal hood; MA = median apophysis; Mt = metatarsus; p = prolateral; PA = patellar apophysis; Pa = patella; PLE = posterior lateral eye; PME = posterior median eye; PME-PLE = distance between PME and PLE; PME-PME = distance between PME and PME; R = receptacle; r = retrolateral; RTA = retrolateral tibial apophysis; ST = subtegulum; T = tegulum; Ta = tarsus; TC = tip of conductor; Ti = tibia; v = ventral; VTA = ventral tibial apophysis. References to figures in the cited papers are listed in lowercase (fig. or figs); figures from this paper are noted with an initial capital (Fig. or Figs).

DNA barcodes were obtained for future use: a partial fragment of the mitochondrial gene cytochrome oxidase subunit I (COI) was amplified and sequenced for these 5 species using primers LCO1490-oono (5’-CWACAAAYCATARRGATATTGG-3’) (Folmer et al. 1994; Miller et al. 2010) and C1-N-2776 (5’-GGATAATCAGAATANCGNCGAGG-3’) (Simon et al. 1994). For additional information on extraction, amplification and sequencing procedures, see Zhao et al. (2013). All sequences were blasted in GenBank; accession numbers are provided in Table 1.

**Table 1.** Voucher specimen information.

Species	GenBank accession number	Sequence length	Collection localities (all in China)
*P. huashanensis* sp. n.	KY593329	1194bp	Shaanxi Prov.: Huayin Prefecture: Mt. Huashan
*P. liansui*	KY593330	1194bp	Hunnan Prov.: Daoxian Co.
*P. lushuiensis* sp. n.	KY593327	1194bp	Yunnan Prov.: Lushui Co.
*P. triglochinata*	KY593328	1194bp	Sichuan Prov.: Mt. Emei
*P. xiyankouensis* sp. n.	KY593331	1194bp	Guangxi Prov.: Yizhou City

All specimens (including molecular vouchers) are deposited in the Institute of Zoology, Chinese Academy of Sciences (IZCAS) in Beijing, China.

## Taxonomy

### Genus Pireneitega

Taxon classificationAnimaliaAraneaeAgelenidae

Kishida, 1955

Pireneitega Kishida, 1955: 11. Type species Amaurobius roscidus L. Koch, 1868 (= P. segestriformis (Dufour, 1820)) from Germany.
Paracoelotes Brignoli, 1982: 348. Type species Coelotes armeniacus Brignoli, 1978 from Turkey.

#### Diagnosis.

**Figure 1.**
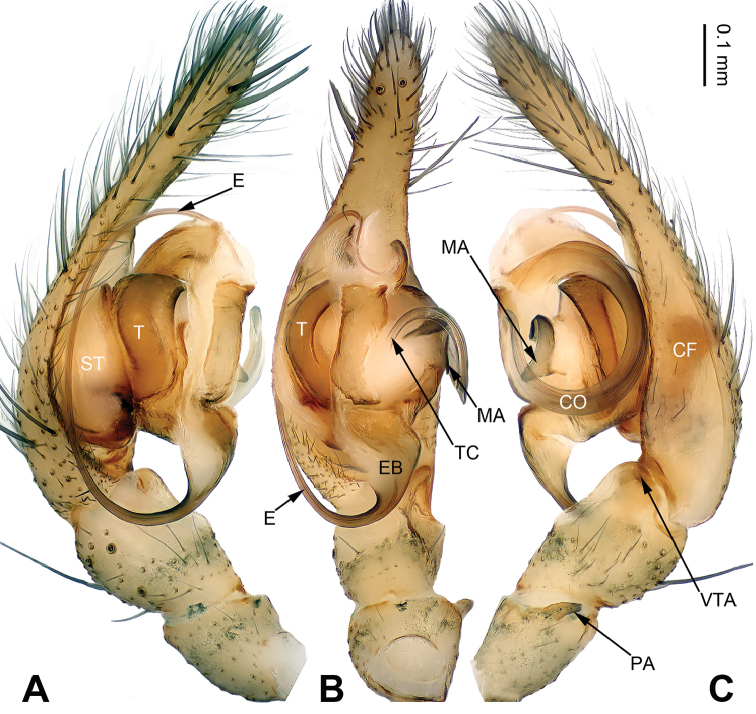
Palp of *Pireneitega huashanensis* sp. n., male holotype. **A** Prolateral view **B** Ventral view **C** Retrolateral view. Scale bar: equal for **A, B, C**.

**Figure 2.**
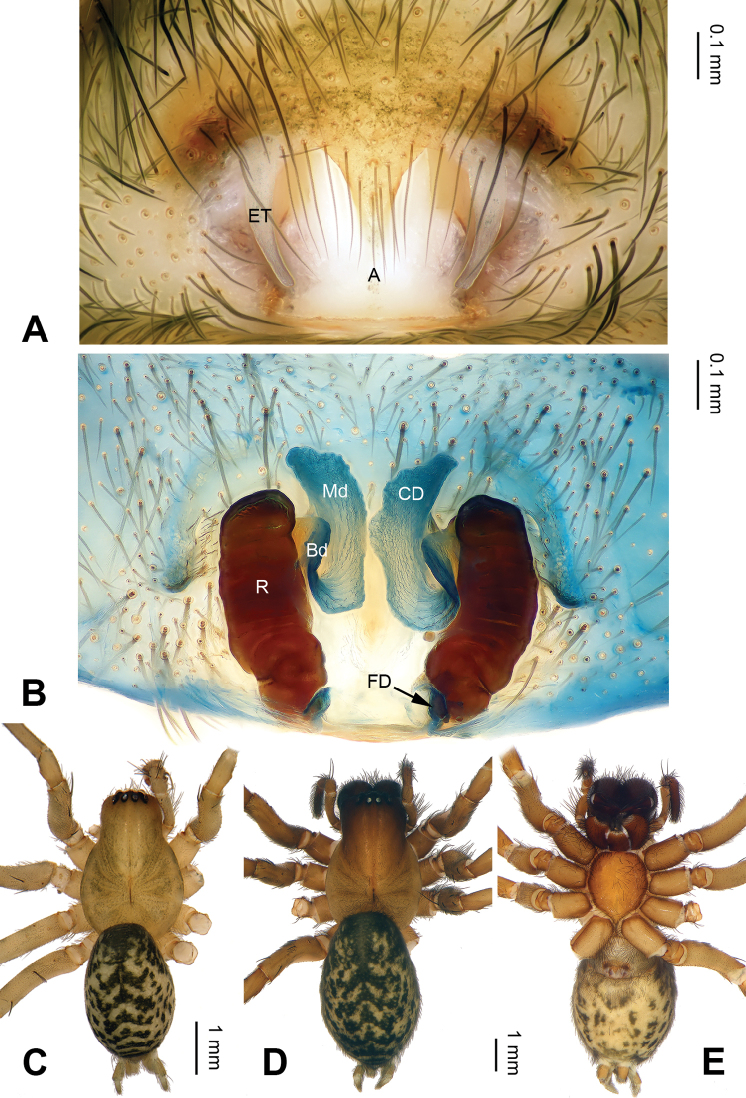
*Pireneitega huashanensis* sp. n., female paratype and male holotype. **A** Epigyne, ventral view **B** Vulva, dorsal view **C** Male habitus, dorsal view **D** Female habitus, dorsal view **E** Female habitus, ventral view. Scale bars: equal for **D, E**.

Females of *Pireneitega* can be distinguished from all other coelotine genera by the widely separated epigynal teeth, the large atrium with subparallel margins, and the broad copulatory ducts (Fig. 2A–B); other coelotines usually have a small atrium and copulatory ducts. The males can be distinguished by the small RTA, the distinct median apophysis and the absence of a conductor dorsal apophysis (Fig. 1A–C); other coelotines usually have a broad conductor dorsal apophysis and a reduced or indistinct median apophysis (Zhang and Marusik 2016).

### Pireneitega huashanensis

Taxon classificationAnimaliaAraneaeAgelenidae

Zhao & Li sp. n.

http://zoobank.org/A75AC3BA-9598-4DEA-B235-1485879B4EFB

Figs 1 , 2 , 11

#### Type material.

**Holotype** ♂: China: ***Shaanxi***: Huayin Prefecture: Mt. Huashan, Duyukou Village, 34°31'42"N, 110°07'22"E, 530 m, 30.IX.2013, Y. Li and J. Liu. **Paratypes**: 1♂, same data as holotype; 4♀1♂, same area, 34°32'46"N, 110°07'06"E, 536 m, 2.X.2016, Z. Zhao and X. Zhang.

#### Etymology.

The specific name refers to the type locality; adjective.

#### Diagnosis.

The male can be distinguished from all other *Pireneitega* species except *P. luniformis* (Zhu & Wang, 1994) by having a tapering conductor tip and longer cymbial furrow. From *P. luniformis*, it can be distinguished by the elongate embolus base and the larger diameter of the conductor’s loop, approximately six times the width of the conductor (*vs* the small embolus base and the small diameter of the conductor’s loop in *P. luniformis*) (Fig. 1; Zhu and Wang 1994: figs 7–8). The female can be distinguished from all other *Pireneitega* species except *P. luniformis* by having short copulatory ducts and long epigynal teeth, subequal to the length of the atrium. From *P. luniformis*, it can be distinguished by the longer septum (*vs* the short septum in *P. luniformis*) (Fig. 2; Zhu and Wang 1994: figs 5–6).

#### Description.

**Male (holotype)**: Total length 5.09. Carapace 2.40 long, 1.85 wide. Abdomen 2.69 long, 1.75 wide. Eye sizes and interdistances: AME 0.13, ALE 0.18, PME 0.15, PLE 0.15; AME-AME 0.03, AME-ALE 0.03, PME-PME 0.05, PME-PLE 0.05. Leg measurements: I: 8.50 (2.50, 2.75, 2.00, 1.25); II: 7.50 (2.25, 2.25, 1.75, 1.25); III: 6.85 (2.00, 2.10, 1.75, 1.00); IV: 9.30 (2.55, 3.00, 2.50, 1.25). Carapace greenish, with black lateral margins, radial grooves indistinct. Abdomen blackish, with yellow herringbone pattern. Palp as in Fig. 1: patellar apophysis short, about four times shorter than tibia; tibia four times shorter than cymbium; VTA long, about 2/3 length of tibia, without pointed tip, extending beyond the tibia; RTA indistinct; cymbial furrow long, more than half the length of cymbium; embolus with broad base, beginning at the 5:30 o’clock position.

Spination in male:

**Female (paratype)**: Total length 8.5. Carapace 3.5 long, 2.9 wide. Abdomen 5.0 long, 2.9 wide. Eye sizes and interdistances: AME 0.16, ALE 0.20, PME 0.16, PLE 0.16; AME-AME 0.10, AME-ALE 0.05, PME-PME 0.10, PME-PLE 0.20. Leg measurements: I: 10.25 (3.00, 3.50, 2.25, 1.50); II: 8.30 (2.50, 3.00, 1.80, 1.00); III: 8.00 (2.40, 2.75, 1.85, 1.00); IV: 11.55 (3.50, 3.75, 3.00, 1.30). Carapace brown. Abdomen black with yellow spots and herringbone pattern. Epigyne as in Fig. 2A–B: epigynal teeth long; septum long with weakly sclerotized tip; atrium with well delimited posterior margin, about 0.6 times longer than wide, about two times longer and wider than septum; copulatory opening distinct; receptacle long, about three times longer than wide, separated by two diameters; copulatory ducts with three parts, the basal part running from receptacle posteriorly (*Bd*), median part running anteriorly (*Md*), and terminal part (*Td*) running posteriorly and leading to copulatory opening; median part as wide as terminal and two times longer than basal part; median part separated; hoods indistinct.

Spination in female:

#### Distribution.

Known only from Shanxi (Fig. 11).

### Pireneitega liansui

Taxon classificationAnimaliaAraneaeAgelenidae

(Bao & Yin, 2004)

Figs 3 , 4 , 11

Coelotes liansui Bao & Yin, 2004: 455, figs 1–3 (♀). Holotype ♀ from Hunan, Daoxian County, 25°31'N, 111°36'E. Types lost (originally at College of Life Science, Hunan Normal University).
Pireneitega liansui : Wang & Jäger 2007: 46 (transfer from Coelotes).
Paracoelotes liansui : Yin et al. 2012: 1020, fig. 528a–c (♀).

#### Material examined.

3♀1♂, China: ***Hunan***: Daoxian County: Dongzhou Village, 25°31'45"N, 111°36'17"E, 168 m, 5.XI.2016, H. Yang.

#### Diagnosis.

**Figure 3.**
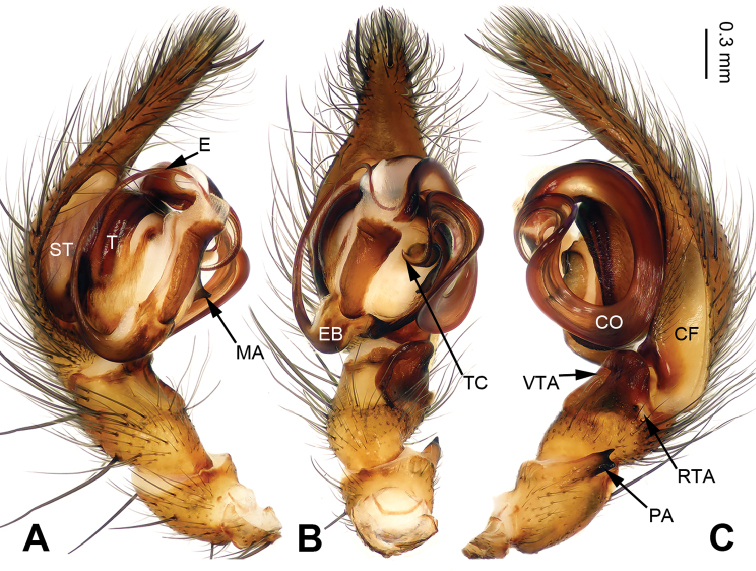
Palp of *Pireneitega liansui*, specimen from Diaoxian. **A** Prolateral view **B** Ventral view **C** Retrolateral view. Scale bar: equal for **A, B, C**.

The male can be distinguished from all other *Pireneitega* species except *P. involuta* (Wang et al., 1990), by having a narrow embolus base and a long cymbial furrow, more than half the length of the cymbium. From *P. involuta* it can be distinguished by the bifurcate tip of the patellar apophysis (*vs* a tapering tip in *P. involuta*) (Fig. 3; Wang et al. 1990: figs 13–15, 18–19).

#### Description.

**Figure 4.**
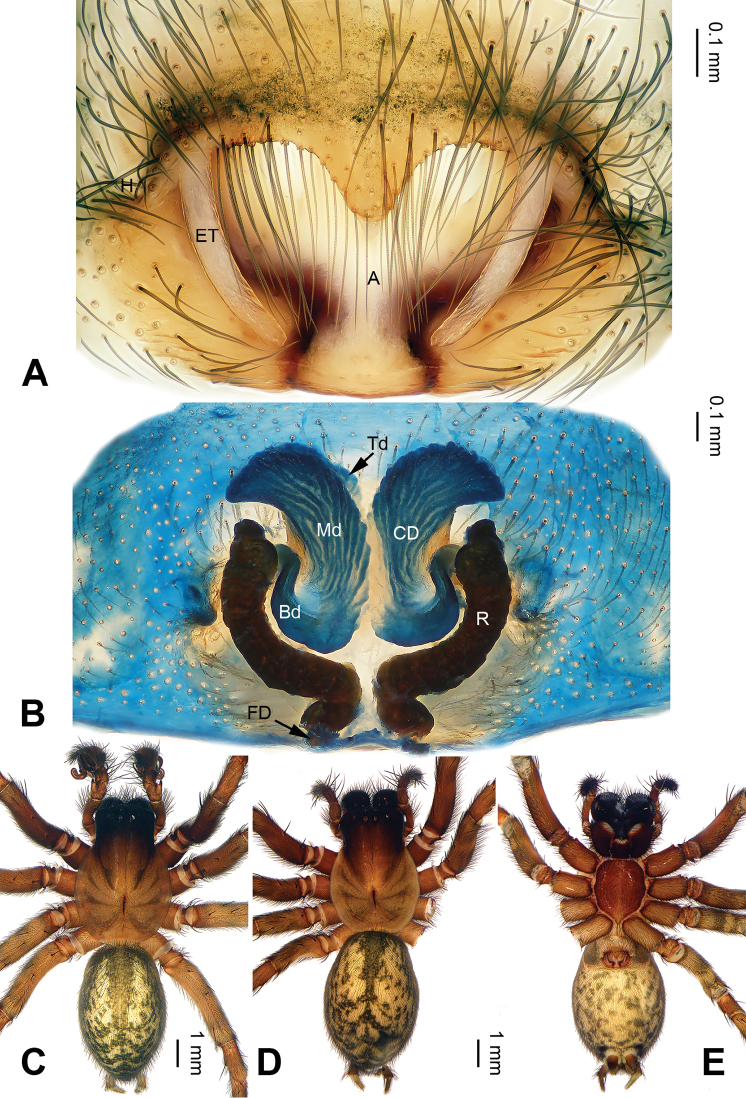
*Pireneitega liansui*, specimens from Diaoxian. **A** Epigyne, ventral view **B** Vulva, dorsal view **C** Male habitus, dorsal view **D** Female habitus, dorsal view **E** Female habitus, ventral view. Scale bars: equal for **D, E**.

**Female** (Fig. 4): Well described by Bao & Yin (2004: figs 1–3).

**Male**: Total length 10.0. Carapace 5.0 long, 3.75 wide. Abdomen 5.0 long, 3.25 wide. Eye sizes and interdistances: AME 0.30, ALE 0.30, PME 0.20, PLE 0.20; AME-AME 0.10, AME-ALE 0.10, PME-PME 0.20, PME-PLE 0.25. Leg measurements: I: 15.75 (4.50, 5.00, 4.00, 2.25); II: 14.45 (4.25, 4.50, 3.70, 20); III: 13.55 (4.00, 4.50, 3.30, 1.75); IV: 17.00 (5.00, 5.50, 4.50, 2.00). Carapace brown, the radial grooves distinct. Abdomen whitish, with green herringbone pattern. Palp as in Fig. 3: patellar apophysis long, about 1/2 length of tibia; tibia short, about four times shorter than cymbium; VTA long, subequal to the tibial length, without pointed tip, extending beyond the tibia; RTA short, about 1/8 length of VTA; width of conductor about 1/5 of loop diameter; embolus beginning at 6:30 o’clock position.

Spination in male:

#### Distribution.

Known only from Hunan (Fig. 11).

#### Remarks.

The male of *P. liansui* is described for the first time.

### Pireneitega lushuiensis

Taxon classificationAnimaliaAraneaeAgelenidae

Zhao & Li sp. n.

http://zoobank.org/25A7D65B-EBAD-486E-81D5-2608E74670D8

Figs 5 , 6 , 11

#### Type material.

**Holotype** ♂: China: ***Yunnan***: Nujiang Lisu Autonomous Prefecture, Lushui County, Pianma Town, 25°59'52"N, 98°37'53"E, 2257 m, 28.VI.2016, Y. Li, M. Xu & M. Hu. **Paratypes**: 8♀5♂, same data as holotype; 3♀2♂, Nujiang Lisu Autonomous Prefecture, Lushui County, 25°59'38"N, 98°39'42"E, 2337 m, 29.VI.2016, Y. Li, M. Xu & M. Hu; 7♀, Baoshan Prefecture, Tengchong City, Mangbang Town, Changlinggan Village, 24°58'07"N, 98°36'54"E, 2032 m, 23.VI.2013, Z. Zhao & J. Liu; 2♀1♂, Baoshan Prefecture, Tengchong City, Mt. Gaoligong National Park, 24°49'44"N, 98°46'03"E, 2177 m, 21–22.VI.2013, Z. Zhao and J. Liu; 10♀, Baoshan Prefecture, Tengchong City, Mingguang Town, Xinjie, Yunyan Temple, 25°29'19"N, 98°32'35"E, 1797 m, 28.XI.2013, Y. Li & J. Liu.

#### Etymology.

The specific name refers to the type locality; adjective.

#### Diagnosis.

**Figure 5.**
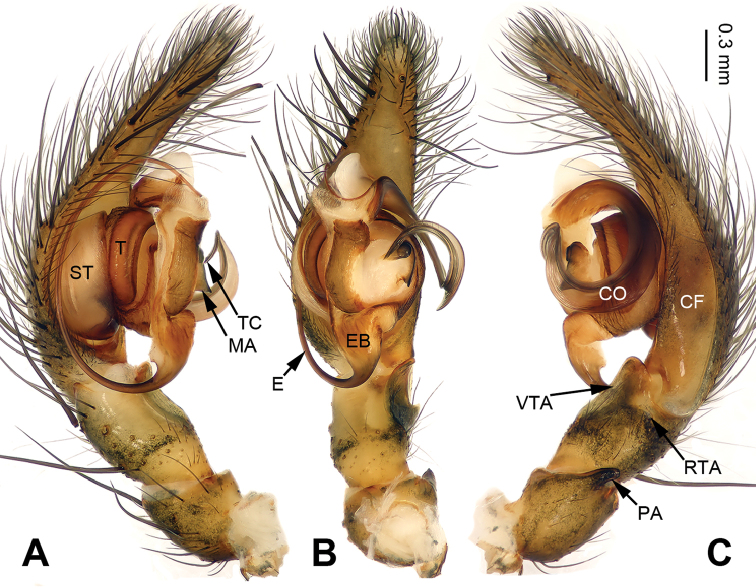
Palp of *Pireneitega lushuiensis* sp. n., male holotype. **A** Prolateral view **B** Ventral view **C** Retrolateral view. Scale bar: equal for **A, B, C**.

**Figure 6.**
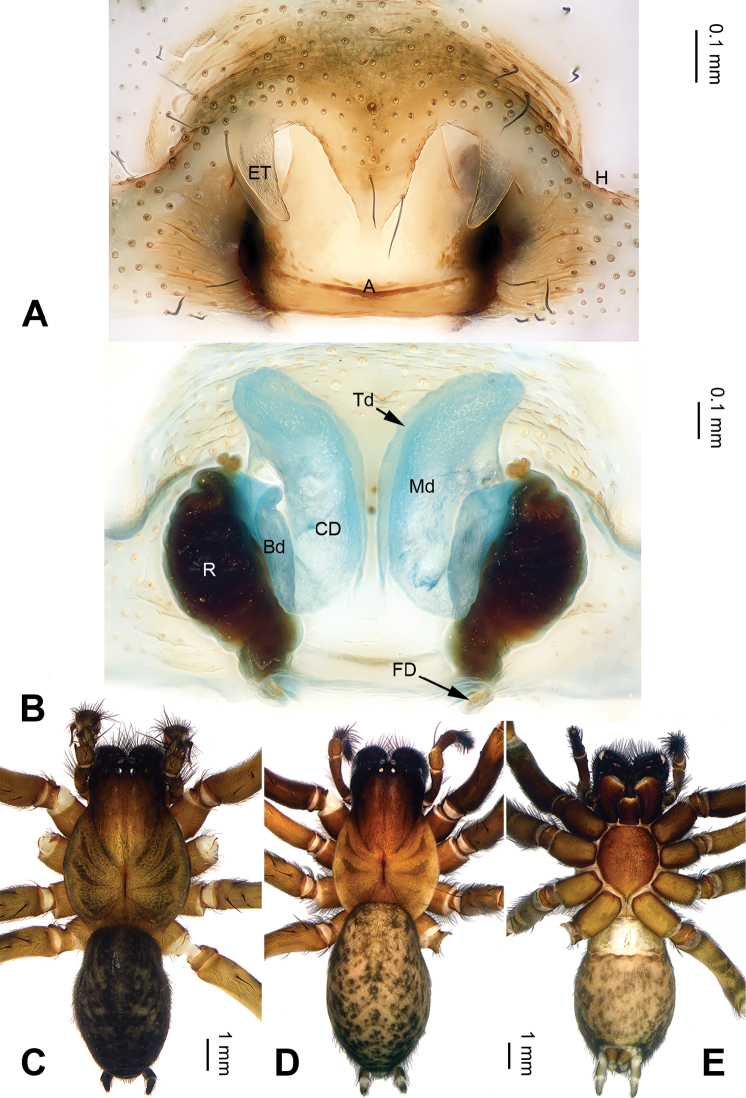
*Pireneitega lushuiensis* sp. n., female paratype and male holotype. **A** Epigyne, ventral view **B** Vulva, dorsal view **C** Male habitus, dorsal view **D** Female habitus, dorsal view **E** Female habitus, ventral view. Scale bars: equal for **D, E**.

The male can be distinguished from all other *Pireneitega* species except *P. huashanensis* and *P. luniformis*, by having a longer cymbial furrow and the arched tip of conductor. From *P. huashanensis* it can be distinguished by the thick tip of the patellar apophysis and the narrow and straight embolus base (*vs* the thin tip of the patellar apophysis and the elongate embolus base in *P. huashanensis*, and the tapering tip of the patellar apophysis, and the small and nearly triangular embolus base in *P. luniformis*) (Figs 1, 5; Zhu & Wang 1994: figs 7–8). The female can be distinguished from all other *Pireneitega* species except *P. luniformis* by having a blunt tip of the septum and a short receptacle. From *P. luniformis* it can be distinguished by long copulatory ducts, and the median part subequal to the length of receptacle (*vs* short copulatory ducts in *P. luniformis*) (Fig. 6; Zhu & Wang 1994: figs 5–6).

#### Description.

**Male (holotype)**: Total length 9.50. Carapace 4.75 long, 3.50 wide. Abdomen 4.75 long, 2.75 wide. Eye sizes and interdistances: AME 0.25, ALE 0.20, PME 0.20, PLE 0.20; AME-AME 0.10, AME-ALE 0.05, PME-PME 0.15, PME-PLE 0.20. Leg measurements: I: 16.25 (4.75, 5.25, 4.00, 2.25); II: 15.00 (4.50, 5.00, 3.50, 2.00); III: 13.00 (4.00, 4.25, 3.00, 1.75); IV: 17.15 (5.00, 5.50, 4.65, 2.00). Carapace yellow with black lateral margins, radial grooves distinct. Abdomen blackish, with gray herringbone pattern. Palp as in Fig. 5: patellar apophysis short, about 1/3 length of tibia; tibia short, about 1/4 length of tarsus; VTA subequal to the tibial length, without pointed tip, extending beyond the tibia; RTA short, about 1/10 length of VTA; cymbial furrow long, more than half length of cymbium; width of conductor about 1/3 of loop diameter; embolus with narrow base originating proximally on base of tegulum, beginning at the 6:00 o’clock position.

Spination in male:

**Female (paratype)**: Total length 10.25. Carapace 4.00 long, 3.25 wide. Abdomen 6.25 long, 3.75 wide. Eye sizes and interdistances: AME 0.25, ALE 0.20, PME 0.20, PLE 0.20; AME-AME 0.10, AME-ALE 0.20, PME-PME 0.20, PME-PLE 0.30. Leg measurements: I: 11.75 (4.00, 4.25, 3.00, 1.50); II: 11.50 (3.50, 4.00, 2.50, 1.50); III: 10.65 (3.35, 3.50, 2.50, 1.30); IV: 14.35 (4.25, 5.00, 3.50, 1.60). Carapace yellow. Abdomen yellow, with black spots and herringbone pattern. Epigyne as in Fig. 6A–B: epigynal teeth broad and long (subequal to length of atrium); septum long with sclerotized tip; atrium with well delimited posterior margin, about two times wider than long, about 1.4 times longer than septum, about 1.8 times wider than septum; copulatory opening distinct; receptacle short, separated by three diameters; copulatory ducts separated, median part as wide as terminal and two times longer than basal part; hoods distinct.

Spination in female:

#### Distribution.

Known only from Yunnan (Fig. 11).

### Pireneitega triglochinata

Taxon classificationAnimaliaAraneaeAgelenidae

(Zhu & Wang, 1991)

Figs 7 , 8 , 11

Coelotes triglochinatus Zhu & Wang, 1991: 1, figs 1–4 (♀ only, male mismatched). Holotype ♀: China: Sichuan: Mt. Emei. Types lost (originally at Jilin University).
Coelotes triglochinatus : Song et al. 1999: 388, f. 225W–X, 227J, 228K (♀ only, male mismatched).
Pireneitega triglochinata : Wang & Jäger 2007: 48 (transfer from Coelotes).

#### Material examined.

China: ***Sichuan***: 2♂, Mt. Emei, Yuanhong Cave, 29°34'08"N, 103°24'32"E, 858 m, 29.IX.2016, Z. Zhao & X. Zhang; 2♀5♂, Mt. Emei, 29°34'11"N, 103°25'36"E, 834 m, 29.IX.2016, Z. Zhao & X. Zhang.

#### Diagnosis.

**Figure 7.**
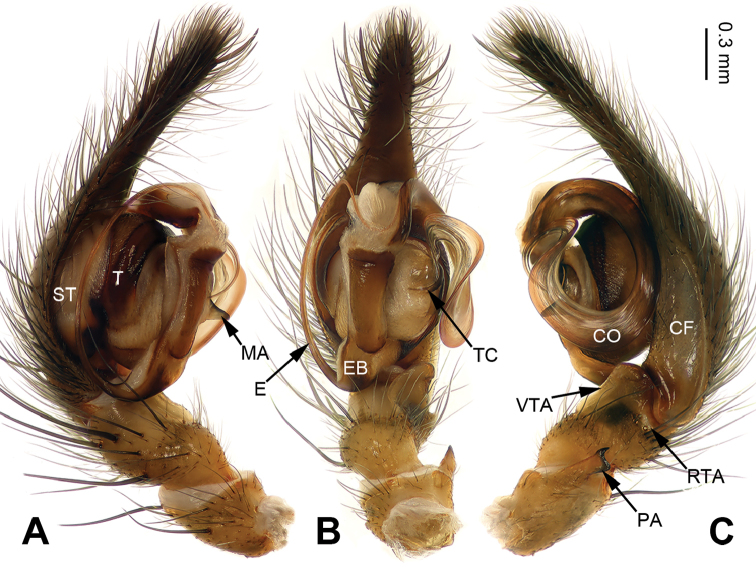
Palp of *Pireneitega triglochinata*, specimen from Mt. Emei. **A** Prolateral view **B** Ventral view **C** Retrolateral view. Scale bar: equal for **A, B, C**.

**Figure 8.**
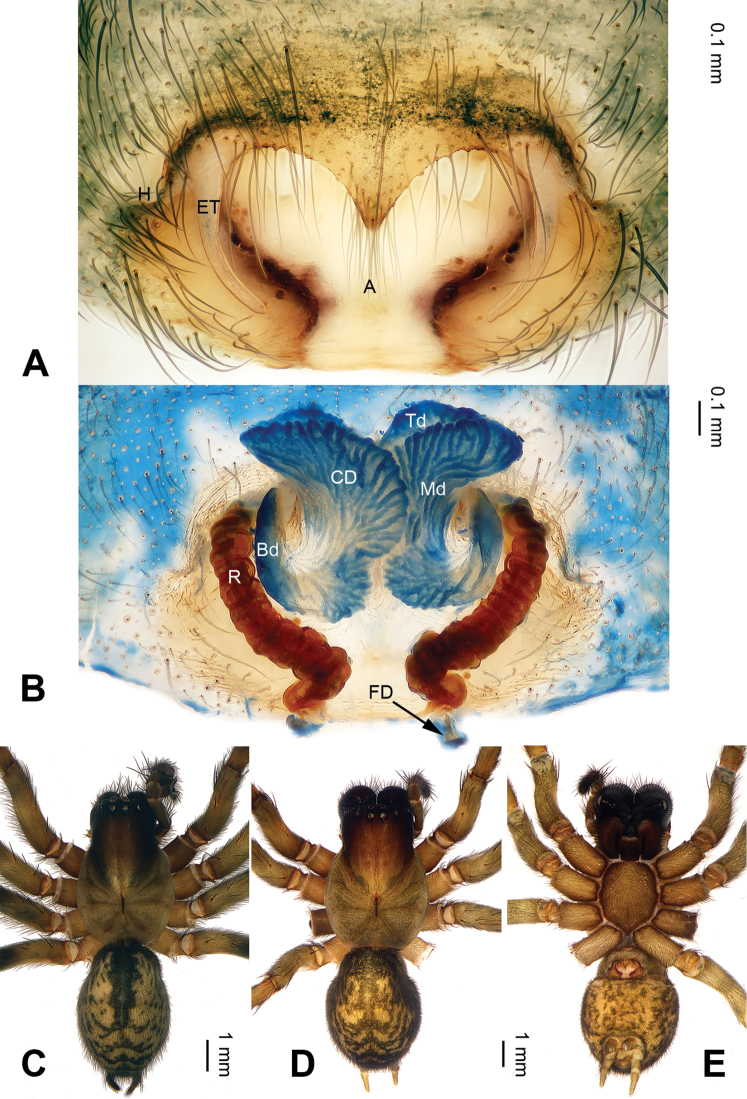
*Pireneitega triglochinata*, specimens from Mt. Emei. **A** Epigyne, ventral view **B** Vulva, dorsal view **C** Male habitus, dorsal view **D** Female habitus, dorsal view **E** Female habitus, ventral view. Scale bars: equal for **D, E**.

The male can be distinguished from all other *Pireneitega* species except *P. involuta* and *P. liansui* by having a broad conductor, the width of the conductor is about 1/5 of the loop diameter. From *P. involuta* it can be distinguished by the embolus base, beginning at the 6:00 o’clock position (*vs* beginning at the 6:30 o’clock position in *P. involuta*). From *P. liansui* it can be distinguished by the tapering tip of the patellar apophysis (*vs* a bifurcate tip in *P. liansui*). (Figs 3, 7; Wang et al. 1990: figs 13–15, 18–19). The female can be distinguished from all other *Pireneitega* species except *P. involuta* and *P. liansui* by having a bent and longer receptacle. From *P. involuta* it can be distinguished by a short septum. From *P. liansui* it can be distinguished by narrow epigynal teeth and the tapering tip of the septum (*vs* broad epigynal teeth and a blunt of septum tip in *P. liansui*) (Figs 4, 8; Wang et al. 1990: figs 16–17).

#### Description.

**Male**: Total length 9.25. Carapace 4.75 long, 3.25 wide. Abdomen 4.50 long, 3.50 wide. Eye sizes and interdistances: AME 0.30, ALE 0.25, PME 0.20, PLE 0.20; AME-AME 0.10, AME-ALE 0.10, PME-PME 0.20, PME-PLE 0.20. Leg measurements: I: 15.75 (4.50, 5.25, 4.00, 2.00); II: 14.00 (4.25, 4.50, 3.50, 1.75); III: 12.45 (3.75, 4.10, 3.00, 1.60); IV: 16.30 (4.75, 5.25, 4.30, 2.00). Carapace brown, radial grooves indistinct. Abdomen yellow with black herringbone pattern. Palp as in Fig. 7: patellar apophysis long, more than half length of tibia, with tapering tip; tibia short, about the same length as cymbium; VTA subequal to the tibial length, without pointed tip, extending beyond the tibia; RTA short, about 1/8 length of VTA; cymbial furrow short, about 1/3 length of cymbium; width of conductor about 1/5 of loop diameter; embolus with broad base, beginning at the 6:00 o’clock position.

Spination in male:

**Female**: Total length 9.75. Carapace 5.00 long, 4.00 wide. Abdomen 4.75 long, 3.50 wide. Eye sizes and interdistances: AME 0.25, ALE 0.20, PME 0.25, PLE 0.20; AME-AME 0.10, AME-ALE 0.10, PME-PME 0.20, PME-PLE 0.25. Leg measurements: I: 14.00 (4.50, 4.75, 3.25, 1.50); II: 13.20 (4.20, 4.50, 3.00, 1.50); III: 12.05 (4.00, 4.00, 2.80, 1.25); IV: 14.50 (4.75, 4.75, 3.50, 1.50). Carapace yellow. Abdomen black with yellow spots and herringbone pattern. Epigyne as in Fig. 8A–B: epigynal teeth narrow and short about 0.9 of atrium length; septum with well sclerotized tip; atrium with weakly delimited posterior margin, about 3.3 times longer than septum, about 1.9 times wider than septum; copulatory opening distinct; receptacle narrow and long, about five times longer than wide, separated by the diameter of receptacle; median part of copulatory ducts as wide as terminal and 1.5 times longer than basal part, median part about three times wider than receptacle; hoods distinct.

Spination in female:

#### Distribution.

Known only from Sichuan (Fig. 11).

#### Note.

The DNA barcode of the male described here matches that of the female. In the original species description of *Coelotes triglochinatus*, the female holotype and male ‘allotype’ were not correctly matched (Wang and Jäger 2007). The male ‘allotype’ of *C. triglochinatus* might match the female of other Coelotinae species described from Mt. Emei. Currently, two Coelotinae species described from Mt. Emei are known only by females, they are *Draconarius sichuanensis* Wang & Jäger, 2007 and *Platocoelotes imperfectus* Wang & Jäger, 2007 (World Spider Catalog 2017).

### Pireneitega xiyankouensis

Taxon classificationAnimaliaAraneaeAgelenidae

Zhao & Li sp. n.

http://zoobank.org/2176DAC7-EF2A-4753-8AEA-153FDE021D35

Figs 9 , 10 , 11

#### Type material.

**Holotype** ♂: China: ***Guangxi***: Hechi Prefecture: Yizhou City: Xiyankou Village, Mt. Baihu, Xiannvyan, 24°29'17"N, 108°34'02"E, 110 m, 11.XII.2012, Z. Chen & Z. Zhao. **Paratypes**: 2♀, same data as holotype; 1♀, Hechi Prefecture: Donglan County: Sanshi Town: Gongping Village, unnamed cave, 24°21'44"N, 107°23'11"E, 383 m, 11.II.2015, Y. Li & Z. Chen; 1♀, Hechi Prefecture: Donglan County: Bala Village, unnamed cave, 24°26'37"N, 107°20'50"E, 385 m, 18.III.2015, Y. Li & Z. Chen; 2♀1♂, Hechi Prefecture: Nandan County: Chengguan Town, unnamed cave, 25°02'11"N, 107°25'00"E, 559 m, 2.II.2015, Y. Li & Z. Chen; 1♀1♂, Chongzuo Prefecture: Daxin County: Fulong Town: Pingliang Village, Banzhongtun, Shuiniu Cave, 22°57'55"N, 107°28'12"E, 248 m, 24.XII.2012, Z. Chen & Z. Zhao; 3♀2♂, Baise Prefecture: Debao County: Yandong Town: Yandong Village, Chuanshan Cave, 23°10'00"N, 106°40'01"E, 596 m, 20.XII.2012, Z. Chen & Z. Zhao; 1♀, Chongzuo Prefecture: Pingxiang City: Liancheng County, Baiyu Cave, 22°07'44"N, 106°45'55"E, 326 m, 28.XII.2012, Z. Chen & Z. Zhao; 1♀, Chongzuo Prefecture: Tiandeng County: Dukang Town: Bakong Village, Yuanliutun, entrance to unnamed cave, 23°06'45"N, 107°04'33"E, 457 m, 26.XII.2012, Z. Chen & Z. Zhao.

**Figure 9.**
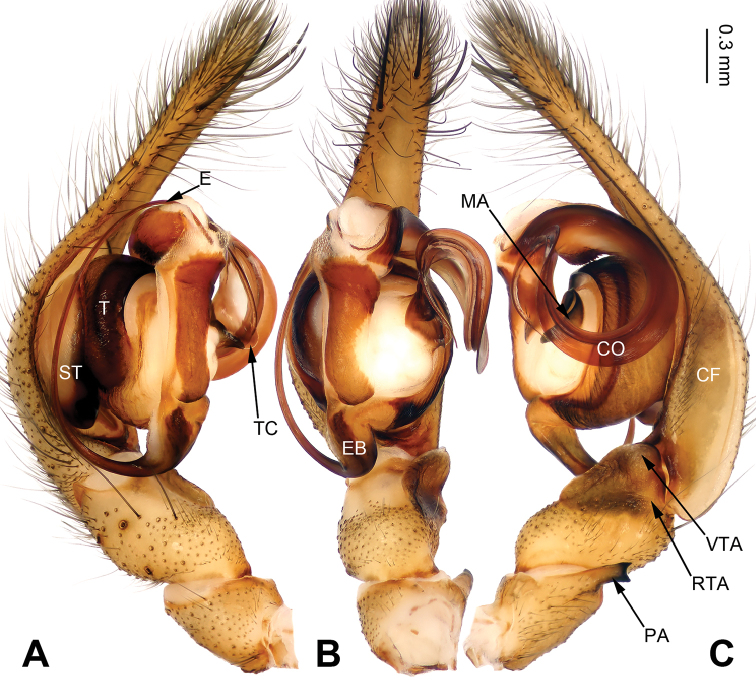
Palp of *Pireneitega xiyankouensis* sp. n., male holotype. **A** Prolateral view **B** Ventral view **C** Retrolateral view. Scale bar: equal for **A, B, C.**

#### Etymology.

The specific name refers to the type locality; adjective.

#### Diagnosis.

**Figure 10.**
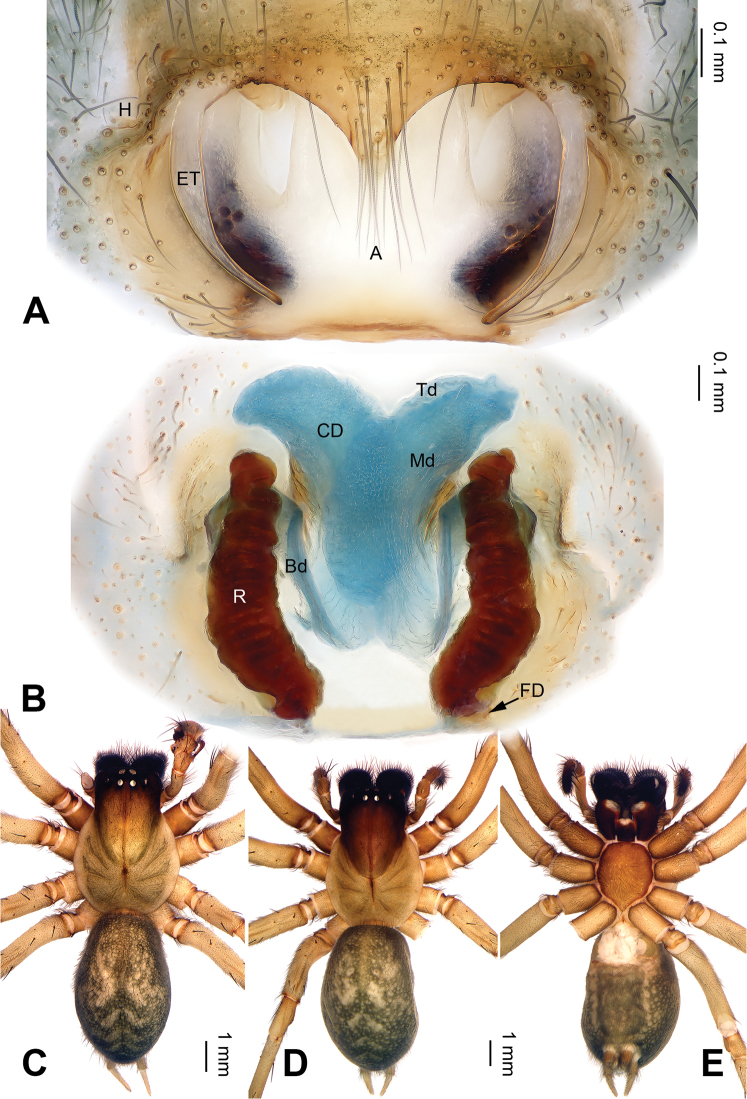
*Pireneitega xiyankouensis* sp. n., female paratype and male holotype. **A** Epigyne, ventral view **B** Vulva, dorsal view **C** Male habitus, dorsal view **D** Female habitus, dorsal view **E** Female habitus, ventral view. Scale bars: equal for **D, E**.

The male can be distinguished from all other *Pireneitega* species except *P. involuta*, *P. liansui* and *P. triglochinata* by having a broad conductor, the width of the conductor about 1/5 of loop diameter. From *P. involuta* it can be distinguished by the bifurcate tip of the patellar apophysis (*vs* a tapering tip in *P. involuta* and *P. triglochinata*). From *P. liansui* it can be distinguished by the short cymbial furrow, about 0.3 times the length of the cymbium (*vs* a long cymbial furrow in *P. liansui*, more than half the length of the cymbium) (Figs 3, 7, 9; Wang et al. 1990: figs 13–15, 18–19). The female can be distinguished from all other *Pireneitega* species except *P. xinping* Zhang, Zhu & Song, 2002 by having bent and narrow epigynal teeth, a broad atrium and sclerotized tip of the septum. From *P. xinping* it can be distinguished by a long receptacle, about four times longer than wide (*vs* a straight and short receptacle in *P. xinping*, about two times longer than wide) (Fig. 10; Zhang et al. 2002: figs 7–8).

#### Description.

**Male (holotype)**: Total length 9.60. Carapace 4.25 long, 3.75 wide. Abdomen 5.35 long, 3.50 wide. Eye sizes and interdistances: AME 0.35, ALE 0.30, PME 0.30, PLE 0.25; AME-AME 0.05, AME-ALE 0.10, PME-PME 0.16, PME-PLE 0.20. Leg measurements: I: 18.85 (5.00, 6.50, 4.85, 2.50); II: 17.25 (4.75, 5.75, 4.50, 2.25); III: 15.70 (4.45, 5.00, 4.25, 2.00); IV: 20.35 (5.50, 6.60, 5.75, 2.50). Carapace yellow, radial grooves distinct, with black lateral margins. Abdomen brown with yellow herringbone pattern. Palp as in Fig. 9: patellar apophysis short, about 1/3 length of tibia; tibia short, about 1/4 length of cymbium; VTA subequal to the tibial length, without pointed tip, extending beyond the tibia; RTA short, about 1/8 length of VTA; cymbial furrow short, about 1/3 length of cymbium; conductor broad; embolus with broad base

Spination in male:

**Female (paratype)**: Total length 10.90. Carapace 5.13 long, 3.95 wide. Abdomen 5.77 long, 3.75 wide. Eye sizes and interdistances: AME 0.35, ALE 0.35, PME 0.26, PLE 0.26; AME-AME 0.10, AME-ALE 0.10, PME-PME 0.24, PME-PLE 0.28. Leg measurements: I: 15.75 (4.50, 5.50, 4.00, 1.75); II: 14.60 (4.25, 5.10, 3.50, 1.75); III: 13.60 (4.10, 4.50, 3.50, 1.50); IV: 16.90 (4.75, 5.65, 4.75, 1.75). Carapace yellow. Abdomen brown with yellow spots and herringbone pattern. Epigyne as in Fig. 10A–B: epigynal teeth narrow and long; septum short with weakly sclerotized tip; atrium with well delimited posterior margin, about three times longer than septum, about 1.3 times wider than septum; copulatory opening distinct; receptacle long, separated by three diameters; median part of copulatory ducts as wide as terminal and 1.3 times longer than basal part, median part about two times wider than receptacle; hoods distinct.

Spination in female:

#### Distribution.

**Figure 11.**
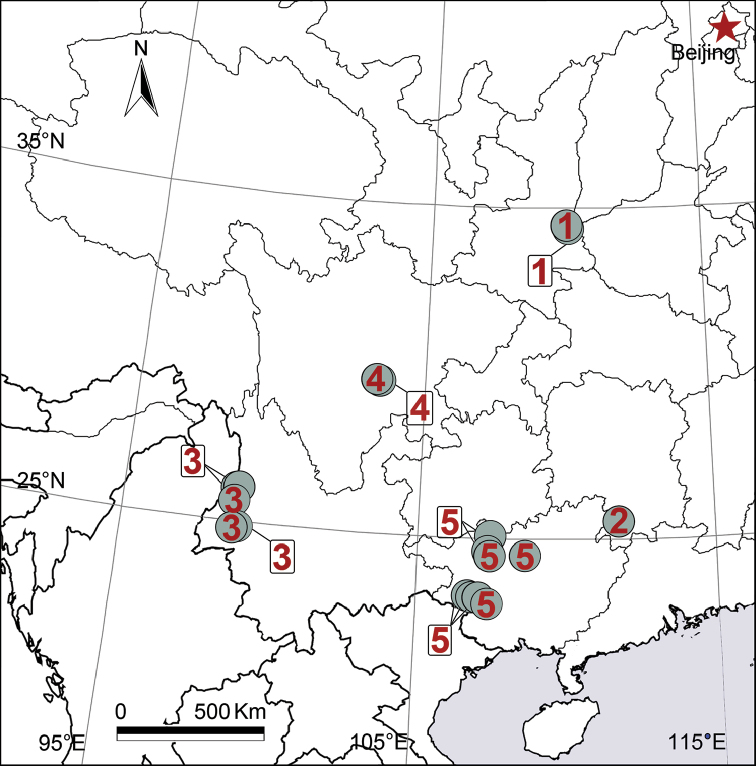
Collection localities of five *Pireneitega* species from China. **1** *P. huashanensis* sp. n. **2** *P. liansui* **3** *P. lushuiensis* sp. n. **4** *P. triglochinata* **5** *P. xiyankouensis* sp. n.

Known only from Guangxi (Fig. 11).

## Supplementary Material

XML Treatment for Pireneitega

XML Treatment for Pireneitega huashanensis

XML Treatment for Pireneitega liansui

XML Treatment for Pireneitega lushuiensis

XML Treatment for Pireneitega triglochinata

XML Treatment for Pireneitega xiyankouensis
